# Population-Based Incidence of Severe Acute Respiratory Virus Infections among Children Aged <5 Years in Rural Bangladesh, June–October 2010

**DOI:** 10.1371/journal.pone.0089978

**Published:** 2014-02-25

**Authors:** Sharifa Nasreen, Stephen P. Luby, W. Abdullah Brooks, Nusrat Homaira, Abdullah Al Mamun, Mejbah Uddin Bhuiyan, Mustafizur Rahman, Dilruba Ahmed, Jaynal Abedin, Mahmudur Rahman, A. S. M. Alamgir, Alicia M. Fry, Peter Kim Streatfield, Anisur Rahman, Joseph Bresee, Marc-Alain Widdowson, Eduardo Azziz-Baumgartner

**Affiliations:** 1 icddr,b, Dhaka, Bangladesh; 2 Centers for Disease Control and Prevention (CDC), Atlanta, Georgia, United States of America; 3 Institute of Epidemiology Disease Control and Research (IEDCR), Dhaka, Bangladesh; Hannover Medical School, Germany

## Abstract

**Background:**

Better understanding the etiology-specific incidence of severe acute respiratory infections (SARIs) in resource-poor, rural settings will help further develop and prioritize prevention strategies. To address this gap in knowledge, we conducted a longitudinal study to estimate the incidence of SARIs among children in rural Bangladesh.

**Methods:**

During June through October 2010, we followed children aged <5 years in 67 villages to identify those with cough, difficulty breathing, age-specific tachypnea and/or danger signs in the community or admitted to the local hospital. A study physician collected clinical information and obtained nasopharyngeal swabs from all SARI cases and blood for bacterial culture from those hospitalized. We tested swabs for respiratory syncytial virus (RSV), influenza viruses, human metapneumoviruses, adenoviruses and human parainfluenza viruses 1–3 (HPIV) by real-time reverse transcription polymerase chain reaction. We calculated virus-specific SARI incidence by dividing the number of new illnesses by the person-time each child contributed to the study.

**Results:**

We followed 12,850 children for 279,029 person-weeks (pw) and identified 141 SARI cases; 76 (54%) at their homes and 65 (46%) at the hospital. RSV was associated with 7.9 SARI hospitalizations per 100,000 pw, HPIV3 2.2 hospitalizations/100,000 pw, and influenza 1.1 hospitalizations/100,000 pw. Among non-hospitalized SARI cases, RSV was associated with 10.8 illnesses/100,000 pw, HPIV3 1.8/100,000 pw, influenza 1.4/100,000 pw, and adenoviruses 0.4/100,000 pw.

**Conclusion:**

Respiratory viruses, particularly RSV, were commonly associated with SARI among children. It may be useful to explore the value of investing in prevention strategies, such as handwashing and respiratory hygiene, to reduce respiratory infections among young children in such settings.

## Introduction

Acute respiratory infections (ARIs) are one of the leading causes of morbidity and mortality among children in low-income countries. In Bangladesh, ARI is a major cause of death among children aged <5 years [1] where approximately 50,000 children annually die from pneumonia [2]. ARI and pneumonia accounted for hospitalization of 40% of 17,815 children aged <5 years during 1997–2001 at five primary level public health care facilities in rural Bangladesh [3]. Most of the known risk factors for pneumonia such as malnutrition, low birth weight, poor air quality, lack of exclusive breast feeding and crowding are prevalent in Bangladesh [2], [4]. Though the ARI burden is well recognized, there is limited information about the etiology-specific incidence of hospitalization for ARI from rural Bangladesh where 70% of the nation’s population lives [5].

During 1988–1989, a study in a rural site of Bangladesh estimated the incidence of ARI to be 5.5 episodes per child-year and the incidence of acute lower respiratory infections (ALRIs) to be 0.2 per child-year among children aged <5 years. Although the etiology for ALRI episodes was not reported, 27 out of 117 (23%) children with ALRI were hospitalized [6]. During July 1999–June 2001, surveillance for ALRIs among a cohort of children aged <5 years in rural Bangladesh estimated the incidence of ALRI-related hospitalization to be 50.2 per 1,000 child-years of observation [7]. This population-based study investigated the bacterial etiology of ALRIs, but no information on viral etiologies was reported. Indeed, before 2004 only limited viral testing was performed in Bangladesh due to lack of molecular assays. Nevertheless, several factors have contributed toward improved laboratory capacity for respiratory viruses in Bangladesh, including the 2005 International Health Regulation and investments in pandemic preparedness [8], [9].

Respiratory virus infections commonly peak during the rainy season in the tropics, but it is unclear which viruses co-circulate during this time of high transmission [10], [11]. Recent data about the etiology-specific incidence of hospitalization from a population-based study may help us to estimate the contribution of viruses to severe respiratory illness. This information can help to determine the need for interventions, and assess whether viral etiology-specific strategies may be a useful approach for prevention of respiratory infections among children aged <5 years in Bangladesh. We conducted a longitudinal study in a rural health and demographic surveillance site of icddr,b to determine the population-based incidence of virus-specific severe acute respiratory infections (SARIs) among community and hospitalized children aged <5 years. One of our primary objectives was to quantify the SARI incidence attributable to influenza during its typical epidemic period in the Bangladesh rainy season [12].

## Methods

### Ethics Statement

The study team obtained written informed consent from the parents of the SARI cases. Icddr,b Institutional Review Board (Research Review Committee and Ethical Review Committee) approved the research protocol. CDC Institutional Review Board reviewed and approved reliance on icddr,b IRB approval.

### Study Site

We conducted our study in Matlab, a rural sub-district in eastern Bangladesh located about 55 km southeast of Dhaka. Since 1966, icddr,b has maintained a health and demographic surveillance system (HDSS) in this community where earlier ARI studies were conducted [6], [7]. The icddr,b surveillance site was divided into two areas; an icddr,b service area in blocks A–D and an area with usual government health services. Each block was comprised of 10–36 villages. The Maternal and Child Health and Family Planning programme provided a range of free services to pregnant women and children aged <5 years in the icddr,b service area through home visits by community health research workers (CHRWs) and through one icddr,b sub-center outpatient medical facility in each block [13]. The icddr,b sub-centers served as the first level of referral. The Matlab hospital run by icddr,b and located in the A block served as the second level of referral. Fee-based government health care facilities were also present in our study areas, which provided health services to all age groups. We conducted this study in all 67 villages of the icddr,b service area and all 12,850 children aged <5 years residing in the area were eligible for inclusion [14]. We assumed that following children in the community and at the icddr,b hospital would allow us to identify the majority of those who developed SARI because during 2009–2010, only 2% of children aged <5 years in the icddr,b service area sought pneumonia care from government health care providers [15].

### Enrollment and Data Collection

We conducted this study during June through October 2010 rainy season. We hired and trained 34 female village health workers to identify potential SARI cases using a checklist for our case-definition (symptoms, age-specific tachypnea and the danger signs). Our case definition for SARI was cough or difficulty breathing with age-specific tachypnea (>60/minute for infant aged 0–1 months, ≥50/minute for infant aged 2–11 months and ≥40/minute for child aged 12–59 months) and one or more danger signs (i.e. unable to drink/breastfeed, lethargic or unconscious, intractable vomiting, convulsions, chest in-drawing or stridor while calm). Through active case detection in the community, we also sought children with cough or difficulty breathing with age-specific tachypnea, without danger signs but still requiring hospitalization. We did not require that children be admitted to the hospital in order to meet the SARI case definition because in the icddr,b service area approximately one in four children aged <5 years were cared for at home rather than being brought to a health care provider for pneumonia care [4].

Health workers made weekly home visit and asked parents about their children’s symptoms and danger signs during the preceding seven days. The field research assistants also made daily visits to the icddr,b Matlab hospital and reviewed the hospital admission record books to identify new SARI cases among hospitalized children. At the end of each day the village health workers and research assistants informed the study physician about potential SARI cases in the community or in hospital via mobile phone. After receiving information about potential SARI cases, the study physician visited the patients at their homes within 48 hours or at the Matlab hospital within 24 hours of notification to confirm that they fulfilled the SARI case definition, enroll cases, and administer a structured questionnaire using a handheld computer. The study physician did not refer community cases to the hospital but advised guardians of all enrolled SARI cases to seek care from an icddr,b subcenter or the Matlab hospital for treatment if they had not done so prior to the physicians’ visit.

The questionnaire contained participants’ demographic and socioeconomic status information (i.e., age, sex, family size, number of rooms in the household, housing materials, ownership of durable goods and monthly household income), where families had sought care for the current SARI event, medical and treatment history (pre-existing medical conditions, symptoms, supplemental oxygen therapy, antibiotics and antivirals), study physician’s physical examination findings (temperature, cyanosis, respiratory rate, breath sounds, rhonchi, crepitation, chest indrawing, stridor and convulsion), peripheral oxygen saturation measured with a portable pulse oximeter and health status at discharge. The study physician collected nasopharyngeal swabs for viral testing from all the enrolled cases and 0.5–3 mls of blood samples using aseptic techniques for bacterial culture from hospitalized cases within 24 hours of admission. We considered it to be a new episode of SARI if the child was symptom free in the preceding 7 days.

### Sample Storage, Transport and Laboratory Analysis

The collected swabs were placed in viral transport media, stored and transported at 2–8°C to the icddr,b virology laboratory on the same day of collection. We tested swabs for RSV, influenza A and B viruses, human metapneumoviruses (HMPV), adenoviruses and human parainfluenza viruses (HPIV) 1–3 by real time reverse transcription polymerase chain reaction (RT-PCR) [primer/probe sequences and assay protocols for non-influenza respiratory viruses available from CDC upon request] [16]. Influenza A positive samples were further characterized for seasonal influenza A(H1N1), A(H3N2), A(H1N1)pdm09 and A(H5N1) using real time RT-PCR [16].

The collected blood samples were inoculated into BacTAlert 3D bottle and then transported at ambient temperature to the icddr,b clinical microbiology laboratory within 24 hours. BacTAlert (Organon Teknika Corp., Durham, N.C) machine positive samples were then sub cultured into Blood agar, MacConkey agar and Chocolate agar. After 24 hours of incubation, the agar plates were observed for the growth of S*treptococcus* species, S*taphylococcus* species, *Hemophilus* species, *Klebsiella pneumoniae*, *Enterococcus faecalis*, *Acinetobacter* species, *Pseudomonas* species and *Candida* species. *Acinetobacter* species was identified using an analytical profile index (bioMérieux, France). We considered growth of coagulase negative *Staphylococcus* as a likely contaminant. Antibiotic susceptibility of the isolated pathogens were performed and interpreted following Clinical and Laboratory Standards Institute guideline [17].

### Follow-up of Hospitalized Cases

The study team called the parents of all hospitalized cases 21 days after hospital discharge to determine the children’s survival status as influenza-associated deaths caused by secondary bacterial infections could occur during this time [18]. We then contacted all the hospitalized cases again at the end of November 2010 (30 days after last SARI case enrollment) to determine if they had recovered completely from their SARI.

### Data Analysis

We performed nonparametric equality-of-median tests and two-sample test of proportions (Z-test) to assess demographic characteristics among hospitalized versus non-hospitalized cases (i.e. children who were cared by their families or by outpatient health providers throughout their illness). To compare the age distribution of hospitalized and non-hospitalized cases with RSV, we performed two-sample Wilcoxon rank-sum (Mann-Whitney) tests. We calculated virus-specific incidences of SARI among hospitalized and non-hospitalized cases by dividing the number of SARI events by the person-time each child contributed to the study. We report the annual incidence (person-years, py) of influenza-associated SARI from five months of data because we assumed that the majority of influenza-associated SARIs occurred during the historical influenza epidemic period [12]. We report the incidence of SARI for other viruses in person-weeks (pw) because we lacked a full year of data. We calculated incidences and 95% confidence intervals (CIs) for incidences using Poisson distribution. We calculated 95% CI for the proportion of blood specimens with a bacterial pathogen using binomial distributions, however, because we collected blood specimens only from hospitalized SARI cases.

## Results

### Profile of Study Participants

**Figure 1:**
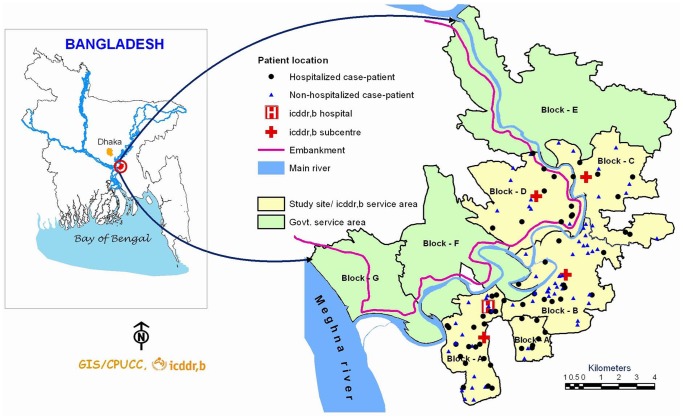
Study site and location of all severe acute respiratory infection cases, Matlab, Bangladesh–2010.

We followed 12,850 children aged <5 years for 279,029 pw (5,337 py), a mean 22 pw per child observed. Of these, 2,528 (20%) children were aged ≤12 months (Table 1). Fifty-one percent of children were male. The study physician identified 141 SARI cases during the study period; 76 (54%) at home (non-hospitalized cases), 58 (41%) in the hospital and 7 (5%) initially identified at home that were later hospitalized (Figure 1). Two children developed two separate episodes of SARI. According to WHO/IMCI criteria [19], 126 (89%) of these 141 SARI cases met the case definition for severe pneumonia or very severe pneumonia.

**Table 1:** Age distribution and follow-up duration of children aged <5 years in Matlab, Bangladesh, June–October 2010**.**

Age group	Population (%),n = 12,850	Male sex,Frequency (%)	Follow-up duration, person-weeks(person-years)
≤12 months	2,528 (20)	1,303 (52)	54,894 (1,050)
<1 month	194 (2)	101 (52)	4,213 (81)
1–2 months	338 (3)	167 (49)	7,339 (140)
3–6 months	851 (7)	435 (51)	18,479 (353)
7–12 months	1145 (9)	600 (52)	24,863 (476)
12–59 months	10, 322 (80)	5,284 (51%)	224,135 (4,287)

### Demographic Characteristics of All Identified SARI Cases

The median age of the hospitalized cases was 9 months (interquartile range, IQR 3–15 months) and the median age of the non-hospitalized cases was 23 months (IQR 11–36 months) (*p*<0.001) (Table 2). Sixty-eight percent of hospitalized cases were male compared to 59% of non-hospitalized cases (*p* = 0.3).

**Table 2:** Demographic characteristics and underlying health status of children aged <5 years with severe acute respiratory infections in Matlab, Bangladesh, June–October 2010.

Characteristics	Frequency (%)		*P*-value
	Hospitalized cases, n = 65	Non-hospitalized cases,n = 76	
Sex, male	44 (68)	45 (59)	0.3
Age in months, median (interquartile range, IQR)	9 (3–15)	23 (11–36)	<0.001*
Pre-existing medical condition†	2 (3)‡	0 (0)	0.1
Number of household members, median (IQR)	5 (4–7)	5 (4–7)	0.9
Number of rooms in the household, median (IQR)	2 (1–3)	2 (2–3)	0.2
>3 people per room in the household (crowding)§	26 (40)	16 (21)	0.01*
Monthly household income (US$), median (IQR)||	96 (71–143)	114 (71–143)	0.2

*P-value for nonparametric equality-of-median test.

† We asked the parent if the case-patient had any medical conditions such as diabetes, chronic heart disease, chronic lung disease, asthma, chronic liver disease, neurologic/neuromuscular disease, hematologic disorder, metabolic disorder, immunosuppressive condition or cancer.

‡ One case had reactive airway disease and the other had congenital heart disease.

§ The median number of household members per 100 sq ft was 2.2 in rural Bangladesh [51].

|| One missing value in each group.

### Laboratory Diagnosis

**Figure 2:**
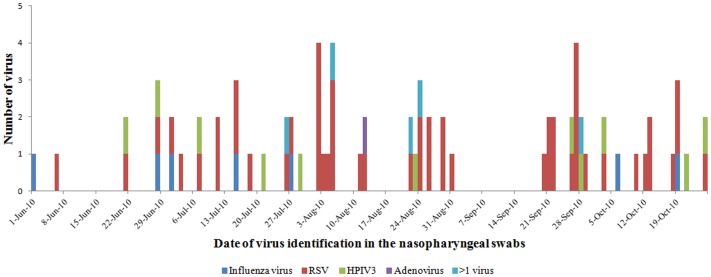
Respiratory viruses identified among children aged <5 years with severe acute respiratory infections during the typical influenza epidemic period in Bangladesh rainy season, 2010.

The median time interval between the first symptom associated with the illness and nasopharyngeal swab collection for hospitalized cases was 4 (IQR 3–5) days and for non-hospitalized cases was 2 (IQR 2–4) days (*p* = 0.005). At least one virus was detected in specimens from 34 of 65 (52%) hospitalized cases and 42 of 76 (55%) non-hospitalized cases (*p* = 0.7). The highest proportion of virus detected throughout the study period was RSV (34% in hospitalized and 39% in non-hospitalized cases, *p* = 0.5) (Table 3) (Figure 2). The median age of hospitalized cases with RSV was lower than non-hospitalized cases (age 7 versus 13 months, p = 0.04). The age-adjusted proportion of hospitalized SARI cases testing positive for individual viruses was similar to that of non-hospitalized cases. We did not identify any influenza A H5 viruses from the SARI cases.

**Table 3:** Respiratory viruses detected from hospitalized and non-hospitalized children aged <5 years with severe acute respiratory infections in Matlab, Bangladesh, June–October 2010.

Virus	Hospitalized cases (N = 65)		Non-hospitalized cases (N = 76)		Total (N = 141)
	n (%, 95% CI)	Age in months, median (IQR)	n (%, 95% CI)	Age in months, median (IQR)	n (%)
RSV	22 (34, 23–47)	7 (3–10)	30 (39, 28–51)	13 (6–24)	52 (37)
HPIV3	6 (9, 3–19)	10 (7–15)	5 (7, 2–15)	12 (7–36)	11 (8)
Influenza viruses	3 (5, 1–13)	15 (9–15)	4 (5, 1–13)	24 (15–30)	7 (5)
Adenoviruses	0 (0, –)	–	1 (1, 0.03–7)	53	1 (1)
HPIV1, HPIV2, HMPV	0 (0, –)	–	0 (0, –)	–	0 (0)
>1 virus	3 (5, 1–13)	4 (1–5)	2 (3, 0.32–9)	23 (20–25)	5 (4)
No virus identified	31 (48, 35–48)	12 (4–27)	34 (45, 33–57)	30 (19–49)	65 (46)

A pathogenic bacteria was isolated from blood cultures of 4 (6% [95% CI 2%–15%]) of 64 hospitalized cases. The pathogens included *Streptococcus* non pneumococcal species, *Enterococcus faecalis, Acinetobacter hemolyticus* and *Candida* species.

### Incidence of SARI and Virus-specific SARI

The overall incidence of SARI was 50.5/100,000 pw (95% CI 42.5–59.6). The highest virus-specific hospitalized SARI incidence was among children aged 1–2 months with RSV (68.1/100,000 pw) followed by HPIV3 (13.6/100,000 pw) (Table 4). The highest virus-specific non-hospitalized SARI incidence was among children aged 3–6 months with RSV (37.9/100,000 pw). The incidence of influenza-associated hospitalization was 1.1/100,000 pw (0.6/1,000 py).

**Table 4:** Incidences of severe acute respiratory virus infections among children aged <5 years in Matlab, Bangladesh, June–October 2010.

Respiratory virus	Incidence (95% CI)					
	<1 month	1–2 months	3–6 months	7–12 months	12–59 months	All ages
**Total SARI**	166.2 (66.8–342.3)	163.5 (84.5–285.6)	102.8 (61.9–160.6)	96.5 (61.9–143.6)	35.3 (27.9–43.9)	50.5 (42.5–59.6)
**Hospitalized cases**						
RSV per 100,000 person-weeks (pw)	47.5 (5.8–171.5)	68.1 (22.1–159.0)	32.5 (11.9–70.7)	16.1 (4.4–41.2)	2.2 (0.7–5.2)	7.9 (4.9–11.9)
HPIV3 per 100,000 pw	−	13.6 (0.3–75.9)	−	12.1 (2.5–35.3)	0.9 (0.1–3.2)	2.2 (0.8–4.7)
Influenza per 100,000 pw	−	−	−	4.0 (0.1–22.4)	0.9 (0.1–3.2)	1.1 (0.2–3.2)
Influenza per 1,000 person-years (py)	−	−	−	2.1 (0.1–11.7)	0.5 (0.1–1.7)	0.6 (0.1–1.6)
Multiple viruses per 100,000 pw	−	13.6 (0.3–75.9)	−	8.0 (1.0–29.1)	−	1.1 (0.2–3.1)
**Non-hospitalized cases**						
RSV per 100,000 pw	−	13.6 (0.3–75.9)	37.9 (15.2–78.1)	24.1 (8.9–52.5)	7.1 (4.1–11.6)	10.8 (7.3–15.4)
HPIV3 per 100,000 pw	−	−	−	12.1 (2.5–35.3)	0.9 (0.1–3.2)	1.8 (0.6–4.2)
Adenovirus per 100,000 pw	−	−	−	−	0.4 (0.0–2.5)	0.4 (0.0–2.0)
Influenza per 100,000 pw	−	−	−	4.0 (0.1–22.4)	1.3 (0.3–3.9)	1.4 (0.4–3.7)
Influenza per 1,000 py	−	−	−	2.1 (0.1–11.7)	0.7 (0.1–2.0)	0.7 (0.2–1.9)
Multiple viruses per 100,000 pw	−	−	−	−	0.9 (0.1–3.2)	0.7 (0.1–2.6)

### Care-seeking, Clinical Features and Treatment of Hospitalized and Non-hospitalized SARI Cases

Overall 51% (72/141) of all identified SARI cases sought care at a hospital or clinic. Of these 72 case-patients, none sought care at the government hospital and 65 sought hospital care at icddr,b, and were hospitalized. Sixty-three percent (41/65) of hospitalized SARI cases only visited icddr,b hospital for treatment while the remaining 37% (24/65) of cases visited two or more health care providers before they were admitted to the icddr,b hospital (Table 5). One-fifth (15/76) of non-hospitalized SARI cases had not visited any health care provider when they were visited by the study physician at a median of 2 days after symptom onset (IQR 1–3 days). The remaining non-hospitalized cases (61/76) visited one or two health care providers for treatment.

**Table 5:** Health care seeking pattern of hospitalized and non-hospitalized children aged <5 years with severe acute respiratory infections in Matlab, Bangladesh, June–October 2010.

Health care sought	Frequency (%)	
	Hospitalized cases, n = 65	Non-hospitalized cases, n = 76
Did not seek care	0 (0)	15 (20)
Visited one health care provider	41 (63)	43 (57)
Matlab hospital	41 (63)	3 (4)
Other hospital/clinic	0 (0)	1 (1)
icddr,b CHRW* or sub-center	0 (0)	17 (22)
Non-icddr,b qualified medical practitioner	0 (0)	1 (1)
Unqualified provider†	0 (0)	21 (28)
Visited two health care providers	20 (31)	18 (24)
Visited three health care providers	3 (5)	0 (0)
Visited four health care providers	1 (2)	0 (0)

*Community Health and Research Worker.

† Includes untrained practitioner, pharmacy/drug seller and homeopath.

Hospitalized cases were admitted a median of 2 days (IQR 1–3) after development of first symptom associated with the illness and were hospitalized for a median of 4 days (IQR 3–6). The predominant signs among hospitalized cases were tachypnoea (88%), rhonchi (83%), crepitation (83%) and chest in-drawing (72%) (Table 6). The predominant signs among non-hospitalized cases were tachypnoea (100%), crepitation (93%) and rhonchi (80%). No danger sign was present in 12% (8/65) of the hospitalized cases when the study physician visited the cases at the hospital. Although non-pneumococcal streptococcus was isolated from a blood culture from one of these eight cases, no viruses were detected from them. Ninety-four percent (61/65) of hospitalized cases received one or more antibiotics and 38% (25/65) of cases received supplemental oxygen. Fifty-nine percent (45/76) of non-hospitalized cases received one or more antibiotics. Fifty-one (78%) cases were discharged from the hospital after improvement of their clinical condition, but were advised to continue treatment at home. During the study period, we identified no cases who died with respiratory symptoms in the community before they could be enrolled and all enrolled cases recovered.

**Table 6:** Clinical features, treatment and status at discharge of the hospitalized children aged <5 years with severe acute respiratory infections in Matlab, Bangladesh, June–October 2010.

Variable	Frequency (%)	
	Hospitalized cases, n = 65	Non-hospitalized cases, n = 76
**Clinical features**		
Tachypnoea	57 (88)	76 (100)
Rhonchi	54 (83)	61 (80)
Crepitation	54 (83)	71 (93)
Subjective fever	50 (77)	65 (86)
Chest in-drawing	47 (72)	47 (62)
Stridor while calm	22 (34)	55 (72)
Mental status change	27 (42)	25 (33)
Unable to drink/breastfeed	19 (30)	21 (28)
Dry cough	10 (15)	3 (4)
Measured temperature ≥100.4°F	5 (8)	2 (3)
Vomiting	5 (8)	7 (9)
Diarrhoea	1 (2)	1 (1)
Convulsion	1 (2)	0 (0)
Cyanosis	1 (2)	0 (0)
Sore throat	0 (0)	2 (3)
Thoracic pain	0 (0)	1 (1)
Myalgia	0 (0)	1 (1)
Any danger sign	57 (88)	63 (83)
Peripheral oxygen saturation (%), median (IQR)	94 (92–98)*	94 (93–97)
**Treatment received**		
Antibiotic agents	61 (94)	45 (59)
Supplemental oxygen	25 (38)	0 (0)
Antiviral agents	0 (0)	0 (0)
Nebulization	42 (65)	2 (3)
Steroid	22 (34)	0 (0)
**Status at discharge**		
Recovered	14 (21)	NA†
Partially recovered‡	51 (79)	
Recovered within 21 days after discharge	49 (96)	
Recovered >21 days after discharge	2 (4)	

*Information available for 62 cases.

† Not Applicable.

‡ Did not recover fully, but with improvement of clinical condition were discharged with advise to continue treatment at home.

## Discussion

Our population-based study findings suggest that young children in this community frequently developed SARI as a result of viral illnesses, in particular RSV, of which only half sought hospital care. Other studies have reported similar findings. RSV was associated with 15–38% of respiratory infections among children aged <5 years visiting healthcare facilities in Vietnam, Thailand, Brazil and Kenya [20]–[23]. Similar to findings from other studies [23], [24], the majority of the RSV hospitalizations in our study were among young infants aged ≤6 months. This is a particularly important age group; globally, children aged 0–11 months hospitalized for SARI had a higher case-fatality ratio than children aged 12–59 months (2.3% versus 0.7%) [25].

Palivizumab, an immunoprophylactic drug recommended in high income countries for children at high risk of complications for RSV, are typically not affordable (USD 1000–5000 per dose) or available in low-income countries like Bangladesh [26], [27]. Although a number of RSV vaccines are currently in development, none are currently approved for use in humans [28]. With the absence of safe and effective RSV vaccines, development of cost-effective RSV-specific prevention and treatment modalities targeted for children would be valuable [29]. Non-pharmaceutical interventions such as promoting handwashing [30] and respiratory hygiene should also be explored as scalable prevention strategies for reducing risks for acquiring respiratory infections in low-income settings. Hand washing proved effective in reducing influenza-like illnesses among school children in Egypt and was associated with prevention of influenza hospitalization among all age groups in Spain [31], [32]. Improving handwashing among hospital staff and visiting families, as well as cohorting children with RSV infections, have been effective in reducing the incidence of nosocomial RSV infection among children aged <2 years in UK [33].

Our study estimated an annual rate of influenza-associated SARI hospitalization based on five months of data collected directly from the population. Despite only 5 months of data collection, our rate is similar to the estimated annual incidences of influenza-associated severe ALRI among children aged <5 years from rural Bangladesh during 1993–1996 [34], and to a low-income urban community in Bangladesh during 2004–2008 [35]. Our rate is within the confidence intervals of the estimated annual incidence of influenza-associated hospitalization among children aged <5 years in the catchment area of 4 selected sentinel hospitals in Bangladesh during 2010 [36] in spite of the different platform and methods used to generate such estimates. Also, our incidence is within the range of the rates of influenza-associated severe ALRI in other low and high-income countries including Kenya, Guatemala, Mozambique, South Africa, El Salvador, Germany, Spain, UK and USA [34] [37]. Estimates of SARI incidences from hospital-based studies, however, are likely to be under-estimates because many SARIs are not hospitalized. For example, in our study 54% of SARI case-patients were not hospitalized and worldwide 38% of children with severe ALRI are never hospitalized [25].

During our five-month study period we identified RSV, HPIV3, influenza viruses and adenoviruses from 52% of the specimens collected from SARI cases. We did not identify any HPIV1 or HPIV2 though HPIV3 was the second most common virus among the SARI cases. We did not detect human metapneumovirus, which has been identified among children aged <5 in Bangladesh in urban and rural communities outside our study period (peaks in April 2001 and November-December 2010) [38], [39]. Other studies focused on ARI or pneumonia conducted for at least 6 months to >3 years have identified similar viruses as we reported in 46–83% of their specimens and also identified human bocavirus, enterovirus, human coronavirus and human rhinoviruses [20], [22], [39], [40]. Additionally, previous studies suggest annual and rural-urban differences in the epidemic period of RSV in Bangladesh [39], [41]. Therefore, our viral yield may have been different if we had collected and tested samples year-round or tested for a greater variety of viruses.

We identified bacterial pathogens from 6% (95%CI 2–15%) of hospitalized cases during our study period. Bacterial pathogens were identified in 95 (11%) of 840 blood samples cultured from hospitalized children with severe ALRIs during July 1999 through June 2001 in our study area [7]. Though we were able to collect blood samples from most of the hospitalized children in this rural setting, the early empirical use of antibiotics likely decreased our ability to detect bacterial pathogens from blood specimens [42], [43]. Even in the absence of antibiotics, blood cultures are insensitive for identifying bacterial pneumonia caused by *Haemophilus influenza* type b and invasive pneumococcal disease [44], [45].

Future studies using newer high-throughput multi-pathogen diagnostics such as multiplex PCRs and other molecular technologies that use samples obtained from the respiratory tract [46], [47], blood PCR [48], and urine assays for pneumococcal antigens [49] in the urine may broaden our understanding of viral and bacterial etiologies of severe respiratory infections. Improved diagnostics may also help formulate preventive strategies and empiric treatment guidelines in low-income settings with limited laboratory capacity and where routine testing of samples for pathogen identification is not currently feasible.

### Limitations

We conducted this study during the June to October 2010 influenza season which limits our capacity to estimate annual incidences of hospitalization for other viruses. Data from this children’s cohort and the national hospital-based influenza surveillance suggests that our study period may have included the majority of influenza infections. Influenza epidemics have been observed in hospital-based influenza surveillance during May-September in 2007–2008 and June–September in 2010 [12], [50]. From the national hospital-based influenza surveillance in Bangladesh, influenza was identified from January through November in 2010 and 63% (357/563) of the influenza illnesses were detected during our study period [50]. Yet, our study included only a proportion of the annual RSV illnesses. In 2010, four surveillance platforms in different locations in Bangladesh suggests that 52% (48/92) of RSV hospitalizations occurred outside our study period (icddr,b internal report). Three to five whole years of data collection could enable us to better calculate virus-specific annual hospitalization rates for all viruses. It is possible that a proportion of children with respiratory infections may have developed complications as a results of their illness, but did not because of their early identification and prompt treatment by icddr,b. The population may also have had increased health awareness and improved health seeking behavior because of periodic visits with our health workers. Therefore, the clinical outcomes of this cohort probably do not reflect those of other areas in Bangladesh. Most of the cases received medications including antibiotics either at a healthcare provider visited prior to hospitalization or immediately after hospitalization before collection of blood sample by the study physician. It was not feasible to collect blood samples from the admitted SARI cases before initiation of medication following hospitalization because field staff sought and identified SARI cases by visiting the hospital only once daily. Thus the study physician collected blood sample within 24 hours of hospitalization.

## Conclusion

Respiratory viruses, and especially RSV, were associated with approximately half of the hospitalizations for severe acute respiratory infection among children aged <5 years in rural Bangladesh during June–October 2010. Bangladesh could benefit from low-cost strategies for the prevention and control of RSV and other respiratory pathogen associated SARI interventions that address multiple risk factors for pneumonia and ALRI; some strategies might include those addressing poor sanitation, malnutrition, low birth weight, air pollution, and crowding [1], [4].
